# Impact of smoking and alcohol consumption on telomere length: A univariable and multivariable Mendelian randomization study

**DOI:** 10.1097/MD.0000000000044685

**Published:** 2025-09-26

**Authors:** Changqiang Feng, Songxin Zhong, Rida Li, Yanni Lin, Jianqing Zhu, Xia Liang, Chao Xiao, Chao Qin

**Affiliations:** a Department of Neurology, The First Affiliated Hospital of Guangxi Medical University, Nanning, Guangxi, China; b Department of Neurology, The Sixth Affiliated Hospital of Guangxi Medical University, Yulin, Guangxi, China.

**Keywords:** alcohol consumption, Mendelian randomization, smoking, telomere length

## Abstract

Telomere length is a potential biomarker for biological aging and is associated with many age-related pathologies. Although observational evidence has linked modifiable lifestyle factors to telomere attrition, the causal relationship between smoking, alcohol consumption, and telomere length remains controversial. This study aimed to investigate the causal effects of smoking and alcohol consumption on telomere length using Mendelian randomization (MR) analysis. Genetic data on smoking initiation (N = 805,431), alcohol consumption (N = 666,978), and telomere length (N = 472,174) were extracted from published summary statistics. Univariable and multivariable MR analyses were performed to comprehensively estimate the independent causal effects of smoking and alcohol consumption on telomere length. Cochran *Q* test and the MR Egger intercept test were used to detect heterogeneity and pleiotropy. A Steiger filtering test was conducted to confirm directional causal effects. Further sensitivity analyses were performed using the leave-one-out method and funnel plots. Inverse variance weighted (IVW) analysis showed that genetically predicted smoking initiation was significantly associated with shorter telomere length (univariable IVW: β = −0.08, 95% confidence interval [CI] = −0.11 to −0.04, *P* < .001; multivariable IVW: β = −0.06, 95% CI = −0.11 to −0.02, *P* = .007). Similarly, genetic predisposition to alcohol consumption was causally associated with shorter telomere length (univariable IVW: β = −0.06, 95% CI = −0.10 to −0.02, *P* = .006; multivariable IVW: β = −0.06, 95% CI = −0.11 to −0.01, *P* = .030). No pleiotropy was identified in either univariable or multivariable MR analyses. The Steiger filtering test validated the accuracy of directional causality (*P* < .001). Finally, leave-one-out analysis and funnel plots further confirmed the robustness and reliability of the findings. This study provides genetic evidence for the causal effects of smoking and alcohol consumption on telomere shortening. Interventions targeting tobacco smoking and alcohol consumption may slow down cellular senescence and mitigate the risk of age-related diseases.

## 1. Introduction

Telomeres are protective structures located at the end of linear chromosomes in eukaryotes, consisting of repetitive deoxyribonucleic acid (DNA) sequences and specific protein complexes.^[1]^ Owing to the end replication problem, telomeres shorten continuously with each cell division, ultimately leading to cellular senescence and apoptosis.^[2]^ Telomere shortening has been reported to be associated with an increased risk of occurrence and development of various age-related diseases, including cardiovascular diseases and cancers.^[3–5]^ Telomere length is mainly determined by genetic factors and also correlates with environmental and lifestyle factors, including smoking and alcohol consumption.^[6–8]^

Smoking and alcohol consumption are modifiable lifestyle factors with potential risk effects. The current evidence regarding the relationship between smoking, alcohol consumption, and telomere length is controversial.^[9,10]^ For instance, a systematic review and meta-analysis incorporating 84 observational studies demonstrated significantly shortened telomere length in tobacco-exposed cohorts compared to nonsmoking controls, with evidence of a dose-dependent relationship persisting across sensitivity analyses.^[11]^ Another 20-year follow-up study revealed a negative correlation between cigarette smoking and telomere length.^[12]^ However, a meta-analysis of 18 longitudinal cohorts showed that smoking does not accelerate telomere attrition.^[13]^ Similar to the effect of smoking on telomere length, conflicting results also exist regarding the association between alcohol consumption and telomere length. Most studies have indicated that moderate alcohol consumption does not affect telomere length.^[14–16]^ While some research have reported an association between alcohol consumption and shorter telomere length.^[17,18]^ In traditional epidemiological studies, causal inference between smoking, alcohol consumption, and telomere length encounters significant methodological constraints, primarily due to intrinsic methodological shortcomings including residual confounding variables, bidirectional temporal associations, and selection bias in participant sampling. Therefore, causal associations between smoking, alcohol consumption, and telomere length remain ambiguous.

Mendelian randomization (MR) studies are rooted in the vast data samples accumulated from genome-wide association studies (GWAS), which is an innovative and alternative methodology for exploring causality in epidemiological research. This approach ingeniously utilizes genetic variations as naturally occurring random experimental tools, enabling precise exploration of potential causal links between exposure factors and outcome events.^[19]^ Recently, many MR studies have elucidated the potential causal roles of smoking and alcohol consumption on various health outcomes.^[20–22]^ Herein, we applied univariable and multivariable MR designs utilizing single nucleotide polymorphisms (SNPs) as instrumental variables to explore the potential causal effects of smoking and alcohol consumption on telomere length.

## 2. Materials and methods

### 2.1. Study design

**Figure 1.**
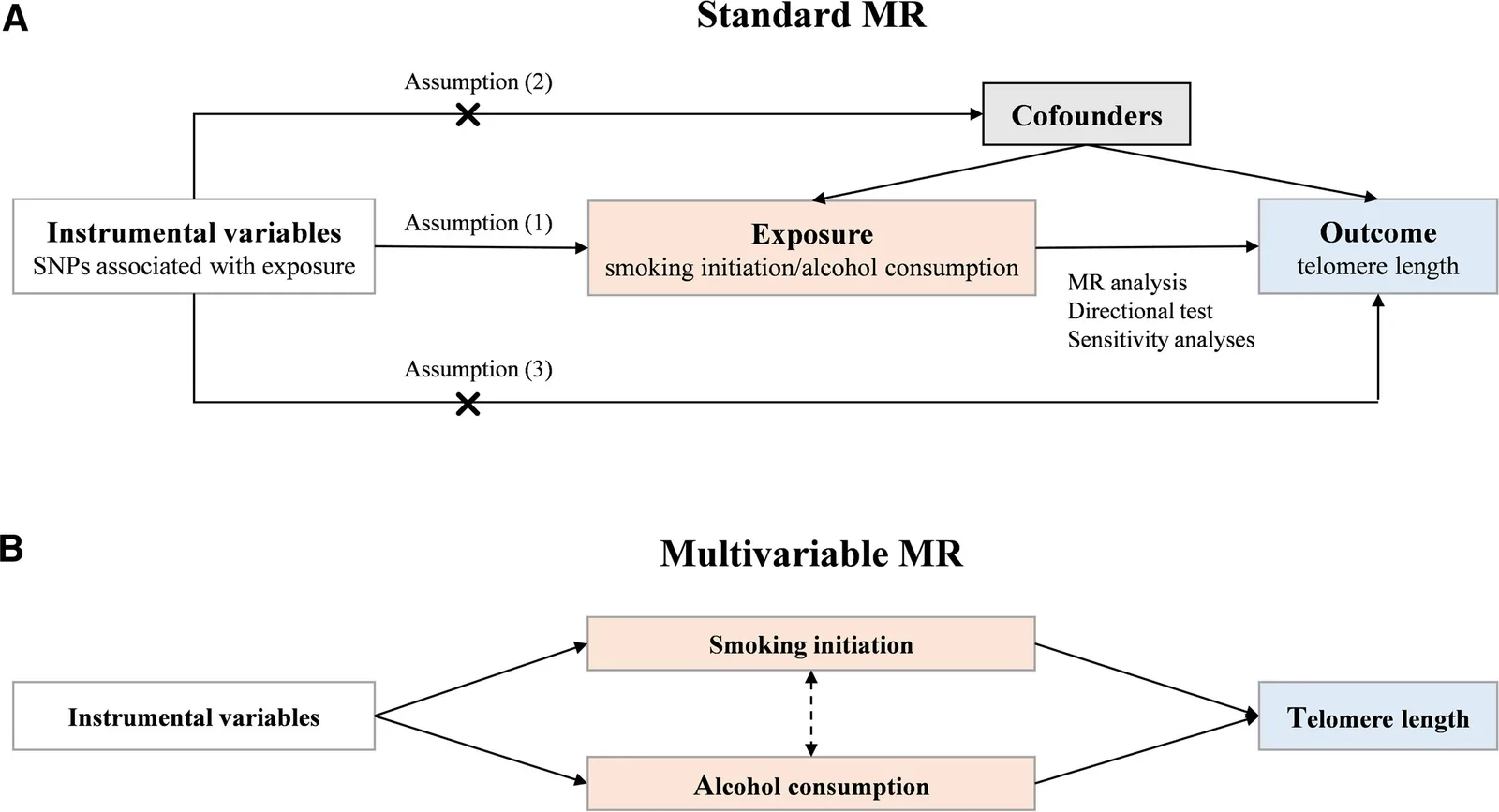
Flow chart of the MR study. (A) Standard MR. MR study relies on 3 core assumptions. Assumption 1: instrumental variables must be strongly correlated with the exposure; Assumption 2: instrumental variables must not be related to any confounders affecting the outcome; Assumption 3: instrumental variables can only impact the outcome through the exposure. (B) Multivariable MR. Genetic instruments associated with smoking and alcohol consumption were utilized to jointly estimate the independent effects of smoking and alcohol consumption on telomere length. MR = Mendelian randomization, SNPs = single nucleotide polymorphisms.

This study defined smoking and alcohol consumption as exposures and telomere length as the outcome, conducting both univariable and multivariable MR analyses using SNPs as instrumental variables. A standard univariable MR study was first performed to comprehensively assess the causal effect of smoking and alcohol consumption on telomere length in the European population (Fig. 1A). Considering the potential genetic association between smoking and alcohol consumption,^[23]^ multivariable MR methods were applied to jointly estimate the independent effects of smoking and alcohol consumption on telomere length (Fig. 1B). This study followed the STROBE-MR reporting guidelines.^[24]^

To avoid sample overlap, genetic data on smoking initiation, alcohol consumption, and telomere length were obtained from 2 different GWAS datasets. Summary statistics for smoking initiation and alcohol consumption were sourced from the largest GWAS meta-analysis performed by the GWAS and Sequencing Consortium of Alcohol and Nicotine use, which included 2669,029 individuals of European ancestry.^[25]^ Smoking initiation was a binary variable defined as ever or currently smoking, and alcohol consumption was a continuous variable referring to the average amount of alcohol consumed per week. Summary statistics for telomere length were extracted from 472,174 European participants in the UK Biobank.^[26]^ Telomere length was a continuous measure expressed as the ratio of telomere repeat copy number to single copy gene, which was determined through a multiplex quantitative polymerase chain reaction method.^[27]^ As this MR study used publicly available data, no ethical approval or informed consent was required.

### 2.2. Instrumental variables selection

As instrumental variables, SNPs must satisfy 3 core assumptions. Assumption 1: SNPs must be strongly correlated with the exposure. Assumption 2: SNPs must not be related to any confounders. Assumption 3: SNPs can only impact the outcome through the exposure. Based on the 3 core assumptions, we first extracted SNPs that were strongly related to smoking initiation and alcohol consumption at a genome-wide significance level of *P* < 5 × 10^−10^. Linkage disequilibrium clumping with *r*^2^ < 0.001 and kb = 10,000 was performed to exclude SNPs with strong linkage disequilibrium. Furthermore, SNPs with *F*-statistics <10 were eliminated to minimize the influence of weak instrumental bias.^[28]^ Subsequently, we employed the LDtrait tool (https://ldlink.nih.gov/?tab=ldtrait) to identify and eliminate SNPs directly associated with telomere length and potential confounding factors (such as body mass index, physical activity, and sleep disorders). Finally, we harmonized the smoking initiation, alcohol consumption and telomere length datasets to ensure the consistency of allele effects (action = 2: Try to infer positive strand alleles, using allele frequencies for palindromes). Before performing MR analysis, MR-PRESSO analysis was conducted to remove outliers with potential pleiotropic effects.^[29]^

### 2.3. MR analysis

The inverse variance weighted (IVW) method was considered to be the most stable and accurate causal relationship method and was usually used as the preferred statistical method for MR analysis.^[30]^ In addition, 2 complementary methods, MR Egger and weighted median, were used to infer causality. The MR Egger method can evaluate whether exposure affects the outcome when all instrumental variables are assumed to be invalid.^[31]^ When 50% of the instrumental variables are invalid, the weighted median method provides a reliable causal estimation.^[32]^ Multivariable IVW and multivariable median methods were used for multivariable MR analysis. These methods rely on different principles; therefore, the consistent effect of multiple methods can draw a more convincing causal conclusion. The MR results were quantified using beta (β) and 95% confidence interval (CI), with a significance set at *P* < .05. Specifically, the causal association was considered meaningful when the IVW result was significant and all methods revealed effects in the same direction.

### 2.4. Sensitivity analysis

Sensitivity analysis was conducted to evaluate the robustness of the MR results. Cochran *Q* test was used to evaluate the heterogeneity among instrumental variables. The MR Egger intercept test was performed to assess potential pleiotropy. A *P*-value <0.05 indicated the existence of heterogeneity or pleiotropy. In addition, we employed the Steiger filtering test to confirm the directional causal effects and exclude the risk of reverse causality.^[33]^ Furthermore, a leave-one-out analysis was performed to investigate whether the causal relationship was driven by a single SNP. The symmetry of the funnel plots could further strengthened the reliability of causal association.

All statistical analyses were performed using the TwoSampleMR (version 0.6.1, University of Bristol, Bristol, UK), MendelianRandomization (version 0.10.0, University of Cambridge, Cambridge, UK) and MR-PRESSO packages (version 1.0, Ron Do laboratory, New York City) in R software (version 4.3.3, University of Bristol, Bristol, UK). Statistical power was calculated using an online tool (https://sb452.shinyapps.io/power/) with a recommended power >80%.

## 3. Result

### 3.1. Instrumental variables

According to rigorous screening criteria, we identified 114 SNPs that served as instrumental variables for smoking initiation and 33 SNPs that served as instrumental variables for alcohol consumption. In multivariable MR analysis, 172 SNPs were selected as instrumental variables. All SNPs demonstrated *F*-statistics >10, indicating no potentially weak instrumental variables (Table S1, Supplemental Digital Content, https://links.lww.com/MD/Q71). The statistical power of smoking initiation (99.1%) and alcohol consumption (83.8%) exceeded 80%, indicating strong reliability of the results.

### 3.2. Casual effect of smoking on telomere length

**Figure 2.**
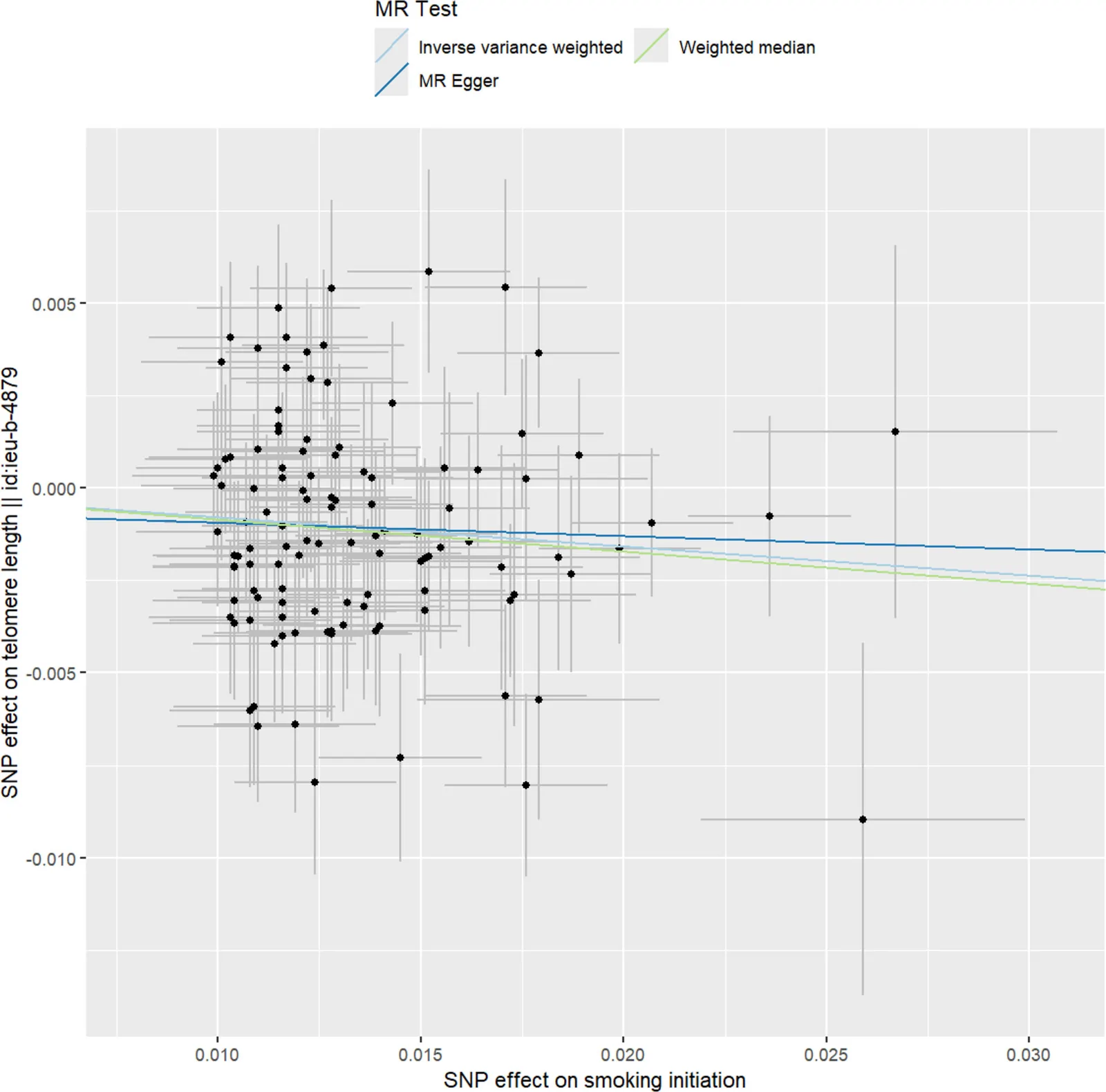
Scatter plots of the causal effect of smoking initiation on telomere length. MR = Mendelian randomization, SNPs = single nucleotide polymorphisms.

The standard MR analysis demonstrated that genetically predicted smoking initiation was significantly associated with shorter telomere length (IVW β = −0.08, 95% CI = −0.11 to −0.04, *P* < .001) (Table 1). In the multivariable MR analysis adjusted for alcohol consumption, the association between genetically predicted smoking initiation and telomere length remained statistically significant (multivariable IVW β = −0.06, 95% CI = −0.11 to −0.02, *P* = .007) (Table 1). In addition, the scatter plots provided a visual representation of the MR analysis and showed consistent directionality (Fig. 2).

**Table 1:** Mendelian randomization results for the effect of smoking initiation and alcohol consumption on telomere length.

Exposure	Outcome	SNPs	Methods	β (95% CI)	*P*
Smoking initiation	Telomere length	114	IVW	−0.08 (−0.11, −0.04)	<.001
			Weighted median	−0.09 (−0.13, −0.04)	<.001
			MR Egger	−0.03 (−0.23, 0.16)	.721
		172	Multivariable IVW	−0.06 (−0.11, −0.02)	.007
			Multivariable median	−0.08 (−0.13, −0.03)	.001
Alcohol consumption	Telomere length	33	IVW	−0.06 (−0.10, −0.02)	.006
			Weighted median	−0.08 (−0.13, −0.03)	.003
			MR Egger	−0.08 (−0.15, −0.02)	.022
		172	Multivariable IVW	−0.06 (−0.11, −0.01)	.030
			Multivariable median	−0.05 (−0.12, 0.01)	.084

CI = confidence interval, IVW = inverse variance weighted, MR = Mendelian randomization, SNPs = single nucleotide polymorphisms.

In the sensitivity analyses, MR Egger intercept revealed no significant pleiotropy (intercept = −5.77E−04, *P* = .662), while Cochran *Q* test showed some degree of heterogeneity (*P*<0.001). Meanwhile, leave-one-out analysis suggested that the causal association between smoking initiation and telomere length was not driven by any single SNP (Fig. S1, Supplemental Digital Content, https://links.lww.com/MD/Q70). Funnel plots showed that the distribution of the SNPs was symmetrical (Fig. S2, Supplemental Digital Content, https://links.lww.com/MD/Q70). Additionally, the Steiger filtering test confirmed the absence of reverse causality (steiger_dir: TRUE, steiger_pval < 0.001), demonstrating the robustness of the results.

### 3.3. Casual effect of alcohol consumption on telomere length

**Figure 3.**
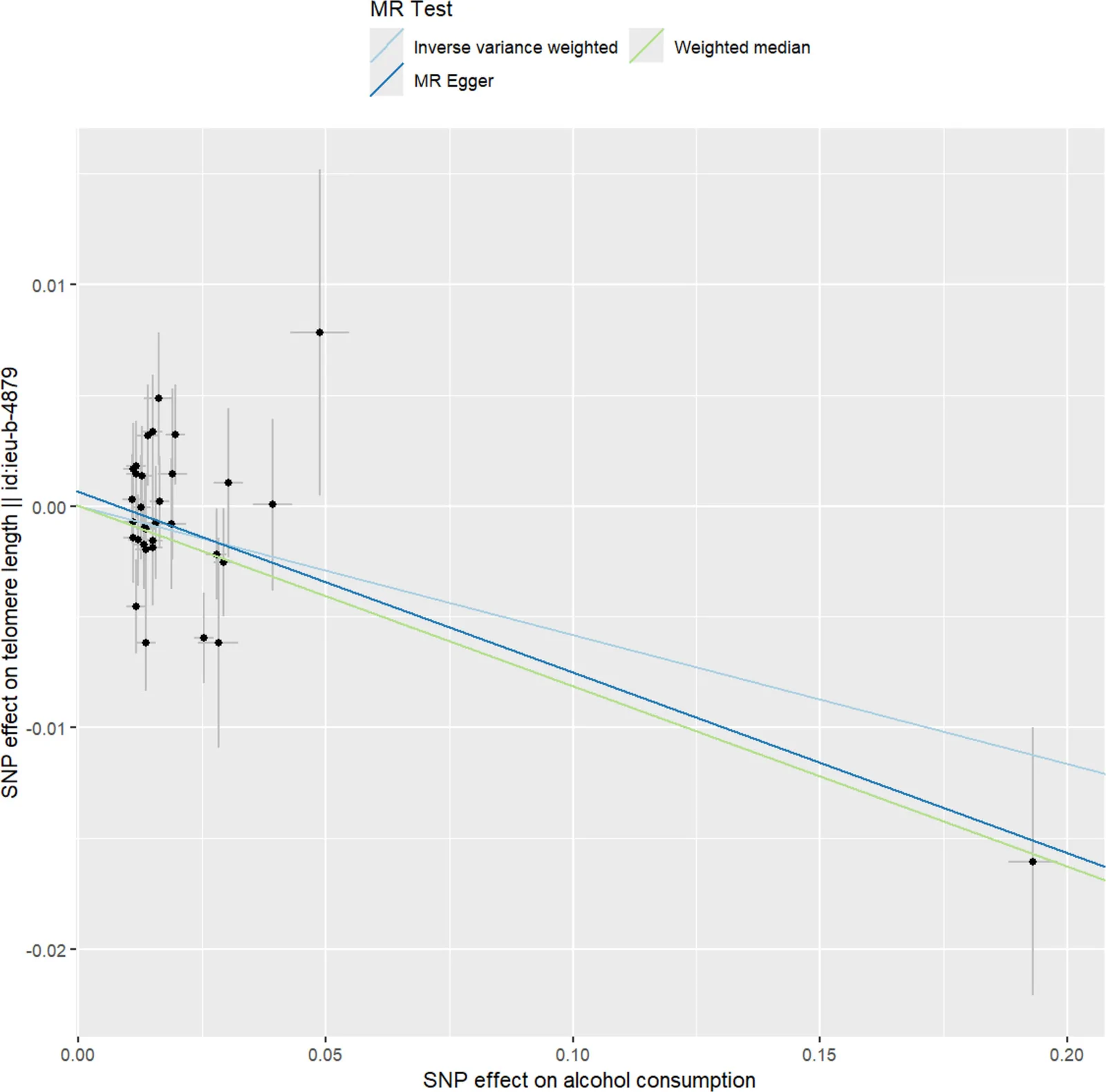
Scatter plots of the causal effect of alcohol consumption on telomere length. MR = Mendelian randomization, SNPs = single nucleotide polymorphisms.

Genetically predicted alcohol consumption was significantly associated with shorter telomere length (IVW β = −0.06, 95% CI = −0.10 to −0.02, *P* = .006) (Table 1). In the multivariable MR analysis, the inclusion of smoking initiation as covariate did not alter the relationship between alcohol consumption and telomere length (multivariable IVW β = −0.06, 95% CI = −0.11 to −0.01, *P* = .030) (Table 1). The scatter plots further visualized the genetic association between alcohol consumption and telomere length (Fig. 3).

In the sensitivity analyses, MR Egger intercept revealed no significant evidence of pleiotropy (intercept = 6.43E−04, *P* = .385), and Cochran *Q* test showed no heterogeneity (*P* = .172). In addition, leave-one-out analysis indicated that no single SNP significantly influenced the results (Fig. S3, Supplemental Digital Content, https://links.lww.com/MD/Q70). Funnel plots showed that the distribution of the SNPs was symmetrical (Fig. S4, Supplemental Digital Content, https://links.lww.com/MD/Q70). Finally, the Steiger filtering test validated the accuracy of the directional causality (steiger_dir: TRUE, steiger_pval < .001), substantiating the robustness of our findings.

## 4. Discussion

Based on large-scale GWAS data, this study systematically investigated the causal effects of smoking and alcohol consumption on telomere length using univariable and multivariable MR methods. Our results indicated that genetically predicted smoking initiation and alcohol consumption were associated with an increased risk of shorter telomere length. Statistical power and comprehensive sensitivity analyses confirmed the robustness and reliability of the findings.

Consistent with most previous studies, we found genetic evidence for a causal relationship between smoking and telomere shortening. The association between smoking and telomere length remained significant even after adjusting for alcohol consumption. This causal association was mechanistically grounded in several underlying biological pathways, including but not limited to exacerbated oxidative stress, chronic inflammatory response, and telomerase activity suppression.^[34,35]^ The oxidative stress mechanism arose from an imbalance between reactive oxygen species production and cellular antioxidant defense systems.^[36]^ Given the high guanine content in telomeric DNA, telomere constituted molecular hotspots for oxidative damage.^[37]^ Chemical components in cigarette smoke could induce an elevation of reactive oxygen species and release inflammatory factors, which directly targeted telomeric regions and accelerated DNA double-strand breaks.^[38,39]^ Meanwhile, continuous tobacco exposure might inhibit telomerase activity and weaken telomere elongation ability.^[7]^ In addition, children born to mothers who smoked during pregnancy demonstrated significantly shorter telomeres compared to nonsmoking controls.^[40,41]^ This indicated that tobacco smoking exhibited transgenerational impacts on telomeric integrity, and prenatal tobacco exposure might elevate offspring susceptibility to accelerated epigenetic aging.

For alcohol consumption and telomere length, our study also strengthened the genetic evidence for the causal effect of alcohol consumption on telomere shortening. In line with a recent MR study, alcohol consumption constituted a significant causal contributor to telomere shortening, underscoring the detrimental impact of alcohol.^[8]^ Similar to cigarette smoking, alcohol metabolism also generated reactive oxygen species and inflammatory factors that induced oxidative stress and inflammatory cascades, precipitating telomeric DNA damage through double-strand breaks and guanine oxidation, ultimately accelerating telomere attrition.^[42,43]^ Furthermore, acetaldehyde – a primary metabolite of alcohol – could induce direct DNA damage and disrupt intracellular metabolic signaling.^[44]^ Crucially, the inhibition of telomerase activity by acetaldehyde impeded the timely repair and elongation of telomere, thereby impairing telomere length maintenance.^[15,45]^ Consequently, alcohol consumption might accelerate telomere attrition through synergistic mechanisms, ultimately driving cellular senescence and age-related pathogenesis.

The principal strength of this investigation resided in the implementation of a more stringent genome-wide significance threshold (*P* < 5 × 10^−10^) for SNPs selection, thereby substantially improving the validity and reliability of genetic instruments for smoking initiation and alcohol consumption exposure analyses. Additionally, the application of comprehensive sensitivity analyses, including pleiotropy test, heterogeneity assessment, directional validation, leave-one-out iterative exclusion analysis, and funnel plot symmetry evaluation, further substantiated the robustness of causal inferences. The primary limitation of this study is that the inclusion of only individuals of European ancestry may restrict the generalizability of the findings to other ethnic groups with distinct lifestyles and socio-cultural contexts. Additionally, the study failed to perform subgroup analyses stratified by demographic factors, such as gender and age. Furthermore, this study aimed to explore the causal relationship between smoking, alcohol consumption and telomere length, and the specific biological mechanisms require further experimental research.

## 5. Conclusion

In conclusion, this study provides genetic evidence for adverse causal effects of smoking and alcohol consumption on telomere length. Interventions targeting tobacco smoking and alcohol consumption may attenuate the attrition rate of telomere, thereby slowing down the cellular senescence cascade and mitigating the risk of age-related diseases. Future studies including in-depth exploration of biological mechanisms and high-quality randomized controlled trials will help to better understand the possible risk factors of telomere length shortening.

## Acknowledgments

We are grateful to the foundations mentioned above and to all the participants in the study.

## Author contributions

**Data curation:** Jianqing Zhu, Xia Liang.

**Methodology:** Changqiang Feng, Rida Li.

**Software:** Songxin Zhong, Yanni Lin.

**Validation:** Rida Li, Jianqing Zhu.

**Visualization:** Yanni Lin, Chao Xiao.

**Writing – original draft:** Changqiang Feng, Songxin Zhong.

**Writing – review & editing:** Changqiang Feng, Songxin Zhong, Chao Qin.

## Supplementary Material
