# Supplementary material for: Impact of smoking and alcohol consumption on telomere length: A univariable and multivariable Mendelian randomization study
Source: Medicine (Baltimore). 2025 Sep 26;104(39):e44685. doi: 10.1097/MD.0000000000044685 (PMC13593225; doi:10.1097/MD.0000000000044685)
Supplement: Supplementary file 1 [file medi-104-e44685-s001.docx]

| Exposure | rsID | EAF | P-value | beta | SE(beta) | N | R2 | F value |
| --- | --- | --- | --- | --- | --- | --- | --- | --- |
| smoking initiation | rs10001365 | 0.378728 | 4.32E-21 | -0.0152 | 0.002 | 805431 | 7.17E-05 | 57.75985657 |
|  | rs10062607 | 0.635189 | 2.28E-10 | 0.0103 | 0.002 | 805431 | 3.29E-05 | 26.52243414 |
|  | rs10233018 | 0.512922 | 1.49E-18 | 0.0139 | 0.002 | 805431 | 6.00E-05 | 48.30238006 |
|  | rs10279261 | 0.629225 | 6.87E-14 | -0.0122 | 0.002 | 805431 | 4.62E-05 | 37.2099076 |
|  | rs10458563 | 0.190855 | 2.17E-18 | 0.0171 | 0.002 | 805431 | 9.08E-05 | 73.10231848 |
|  | rs1050847 | 0.550696 | 3.71E-11 | -0.0105 | 0.002 | 805431 | 3.42E-05 | 27.56243156 |
|  | rs10753630 | 0.348907 | 9.96E-11 | 0.0109 | 0.002 | 780334 | 3.81E-05 | 29.70242387 |
|  | rs10786721 | 0.416501 | 7.91E-27 | 0.0179 | 0.002 | 748334 | 1.07E-04 | 80.10228592 |
|  | rs1084445 | 0.225646 | 1.80E-10 | -0.0121 | 0.002 | 805431 | 4.54E-05 | 36.60240911 |
|  | rs10927039 | 0.196819 | 2.39E-10 | -0.0127 | 0.002 | 805431 | 5.01E-05 | 40.32239987 |
|  | rs11012726 | 0.33002 | 4.25E-17 | 0.0143 | 0.002 | 805431 | 6.35E-05 | 51.12237306 |
|  | rs11078713 | 0.408549 | 2.77E-15 | -0.0126 | 0.002 | 805431 | 4.93E-05 | 39.68990144 |
|  | rs11103667 | 0.178926 | 1.47E-10 | 0.0129 | 0.002 | 805431 | 5.16E-05 | 41.6023967 |
|  | rs111861749 | 0.124254 | 3.16E-12 | -0.0179 | 0.003 | 805431 | 4.42E-05 | 35.60102271 |
|  | rs11210229 | 0.564612 | 4.94E-18 | -0.014 | 0.002 | 805431 | 6.08E-05 | 48.99987833 |
|  | rs11258417 | 0.335984 | 3.83E-10 | -0.0101 | 0.002 | 805431 | 3.17E-05 | 25.50243667 |
|  | rs1126757 | 0.472167 | 4.15E-11 | 0.0104 | 0.002 | 805431 | 3.36E-05 | 27.03993286 |
|  | rs11626595 | 0.156064 | 2.73E-12 | -0.0151 | 0.002 | 805431 | 7.08E-05 | 57.00235845 |
|  | rs11656151 | 0.200795 | 5.61E-12 | -0.0128 | 0.002 | 805431 | 5.09E-05 | 40.95989829 |
|  | rs11716705 | 0.267396 | 1.40E-10 | 0.0117 | 0.002 | 805431 | 4.25E-05 | 34.22241502 |
|  | rs11720703 | 0.506958 | 5.58E-12 | 0.0109 | 0.002 | 805431 | 3.69E-05 | 29.70242624 |
|  | rs11742625 | 0.115308 | 2.19E-10 | 0.0157 | 0.002 | 805431 | 7.65E-05 | 61.62234698 |
|  | rs11873164 | 0.131213 | 1.63E-12 | -0.0155 | 0.002 | 805431 | 7.46E-05 | 60.06235086 |
|  | rs12027999 | 0.151093 | 1.54E-13 | -0.0184 | 0.002 | 732296 | 1.16E-04 | 84.63976884 |
|  | rs12036050 | 0.284294 | 7.18E-11 | 0.0115 | 0.002 | 805431 | 4.10E-05 | 33.0624179 |
|  | rs12112638 | 0.234592 | 8.74E-12 | -0.0122 | 0.002 | 805431 | 4.62E-05 | 37.2099076 |
|  | rs12133063 | 0.371769 | 1.15E-12 | 0.0116 | 0.002 | 805431 | 4.18E-05 | 33.63991647 |
|  | rs12441907 | 0.166998 | 6.09E-14 | -0.015 | 0.002 | 805430 | 6.98E-05 | 56.24986032 |
|  | rs1246265 | 0.701789 | 1.27E-11 | 0.0116 | 0.002 | 805431 | 4.18E-05 | 33.63991647 |
|  | rs12642744 | 0.757455 | 5.98E-11 | -0.0124 | 0.002 | 748334 | 5.14E-05 | 38.43989727 |
|  | rs12685816 | 0.150099 | 1.66E-10 | 0.0145 | 0.002 | 781945 | 6.72E-05 | 52.56236556 |
|  | rs12742446 | 0.630219 | 2.74E-10 | 0.0119 | 0.002 | 630626 | 5.61E-05 | 35.40238772 |
|  | rs1291865 | 0.531809 | 2.12E-13 | 0.0116 | 0.002 | 805431 | 4.18E-05 | 33.63991647 |
|  | rs12923427 | 0.219682 | 4.96E-12 | -0.0136 | 0.002 | 805431 | 5.74E-05 | 46.23988518 |
|  | rs13030994 | 0.508946 | 2.42E-39 | 0.0207 | 0.002 | 805431 | 1.33E-04 | 107.122234 |
|  | rs13255625 | 0.695825 | 4.03E-12 | 0.0116 | 0.002 | 805431 | 4.18E-05 | 33.63991647 |
|  | rs13301073 | 0.348907 | 3.13E-11 | 0.0109 | 0.002 | 805431 | 3.69E-05 | 29.70242624 |
|  | rs1334557 | 0.245527 | 1.51E-12 | 0.0132 | 0.002 | 805431 | 5.41E-05 | 43.55989183 |
|  | rs134529 | 0.379722 | 8.86E-13 | -0.0116 | 0.002 | 805431 | 4.18E-05 | 33.63991647 |
|  | rs1381287 | 0.50497 | 7.51E-15 | 0.0123 | 0.002 | 805431 | 4.70E-05 | 37.82240608 |
|  | rs1435679 | 0.581511 | 2.86E-10 | 0.01 | 0.002 | 805430 | 3.10E-05 | 24.99993792 |
|  | rs143909875 | 0.107356 | 3.74E-12 | 0.0176 | 0.003 | 805431 | 4.27E-05 | 34.41769231 |
|  | rs1503211 | 0.454274 | 3.43E-10 | 0.00999 | 0.002 | 805431 | 3.10E-05 | 24.94996305 |
|  | rs1733756 | 0.49503 | 1.23E-10 | 0.0101 | 0.002 | 805431 | 3.17E-05 | 25.50243667 |
|  | rs1971318 | 0.129225 | 7.80E-13 | 0.0156 | 0.002 | 805431 | 7.55E-05 | 60.83984893 |
|  | rs1994247 | 0.514911 | 1.25E-14 | 0.0122 | 0.002 | 805431 | 4.62E-05 | 37.2099076 |
|  | rs2072155 | 0.699801 | 1.16E-13 | 0.0128 | 0.002 | 805431 | 5.09E-05 | 40.95989829 |
|  | rs2163413 | 0.175944 | 3.06E-11 | -0.0138 | 0.002 | 796935 | 5.97E-05 | 47.60988052 |
|  | rs2289791 | 0.240557 | 2.87E-12 | -0.0128 | 0.002 | 805430 | 5.09E-05 | 40.95989829 |
|  | rs2292239 | 0.666998 | 1.03E-10 | 0.0108 | 0.002 | 805431 | 3.62E-05 | 29.15992759 |
|  | rs2313500 | 0.244533 | 1.99E-12 | 0.0128 | 0.002 | 805431 | 5.09E-05 | 40.95989829 |
|  | rs2678903 | 0.591451 | 1.25E-10 | 0.0104 | 0.002 | 805431 | 3.36E-05 | 27.03993286 |
|  | rs2939756 | 0.489066 | 6.87E-11 | -0.0103 | 0.002 | 805431 | 3.29E-05 | 26.52243414 |
|  | rs3213876 | 0.315109 | 5.08E-12 | 0.0115 | 0.002 | 805431 | 4.10E-05 | 33.0624179 |
|  | rs34367058 | 0.170974 | 1.36E-13 | 0.0138 | 0.002 | 805431 | 5.91E-05 | 47.60988178 |
|  | rs34488670 | 0.206759 | 8.73E-20 | 0.0176 | 0.002 | 805430 | 9.61E-05 | 77.43980771 |
|  | rs3781295 | 0.387674 | 1.13E-12 | -0.0116 | 0.002 | 805431 | 4.18E-05 | 33.63991647 |
|  | rs3801289 | 0.363817 | 3.64E-12 | -0.0115 | 0.002 | 805431 | 4.10E-05 | 33.0624179 |
|  | rs3895907 | 0.548708 | 1.56E-14 | -0.0129 | 0.002 | 723237 | 5.75E-05 | 41.60238495 |
|  | rs3905125 | 0.542744 | 4.28E-11 | 0.0104 | 0.002 | 805431 | 3.36E-05 | 27.03993286 |
|  | rs3934797 | 0.171968 | 1.90E-13 | -0.0151 | 0.002 | 805431 | 7.08E-05 | 57.00235845 |
|  | rs39784 | 0.722664 | 6.41E-11 | 0.0115 | 0.002 | 805431 | 4.10E-05 | 33.0624179 |
|  | rs404263 | 0.65507 | 1.55E-10 | -0.0112 | 0.002 | 697342 | 4.50E-05 | 31.35991006 |
|  | rs4044321 | 0.668986 | 3.04E-23 | -0.0164 | 0.002 | 805431 | 8.35E-05 | 67.23983303 |
|  | rs4374330 | 0.764414 | 3.73E-12 | 0.0127 | 0.002 | 805431 | 5.01E-05 | 40.32239987 |
|  | rs4543592 | 0.49006 | 2.51E-14 | 0.012 | 0.002 | 805431 | 4.47E-05 | 35.99991061 |
|  | rs4571506 | 0.481113 | 1.64E-21 | -0.0151 | 0.002 | 805431 | 7.08E-05 | 57.00235845 |
|  | rs4579569 | 0.499006 | 1.23E-14 | 0.0122 | 0.002 | 805431 | 4.62E-05 | 37.2099076 |
|  | rs465646 | 0.849901 | 1.70E-28 | -0.0236 | 0.002 | 805431 | 1.73E-04 | 139.2396542 |
|  | rs4785187 | 0.244533 | 2.03E-10 | 0.0119 | 0.002 | 805431 | 4.40E-05 | 35.40241209 |
|  | rs4944844 | 0.84493 | 2.33E-10 | -0.0133 | 0.002 | 805431 | 5.49E-05 | 44.22239019 |
|  | rs540356 | 0.370775 | 4.07E-13 | 0.0116 | 0.002 | 805431 | 4.18E-05 | 33.63991647 |
|  | rs540860 | 0.562624 | 3.18E-13 | 0.0117 | 0.002 | 780334 | 4.39E-05 | 34.22241229 |
|  | rs55921136 | 0.240557 | 1.90E-12 | -0.0136 | 0.002 | 805431 | 5.74E-05 | 46.23988518 |
|  | rs55942317 | 0.0795229 | 4.08E-11 | 0.0173 | 0.003 | 805431 | 4.13E-05 | 33.25436187 |
|  | rs56820925 | 0.354871 | 2.25E-10 | -0.0108 | 0.002 | 744041 | 3.92E-05 | 29.15992162 |
|  | rs574835 | 0.365805 | 2.33E-10 | -0.0103 | 0.002 | 805431 | 3.29E-05 | 26.52243414 |
|  | rs5751239 | 0.527833 | 1.02E-10 | -0.0102 | 0.002 | 805431 | 3.23E-05 | 26.00993541 |
|  | rs58400863 | 0.385686 | 5.03E-12 | -0.0114 | 0.002 | 805431 | 4.03E-05 | 32.48991932 |
|  | rs6088618 | 0.4334 | 5.89E-11 | -0.0108 | 0.002 | 744041 | 3.92E-05 | 29.15992162 |
|  | rs6141314 | 0.215706 | 3.59E-13 | 0.0131 | 0.002 | 801138 | 5.35E-05 | 42.9023929 |
|  | rs61533748 | 0.377734 | 1.50E-10 | 0.0104 | 0.002 | 805431 | 3.36E-05 | 27.03993286 |
|  | rs61959481 | 0.203777 | 2.06E-10 | -0.0123 | 0.002 | 805431 | 4.70E-05 | 37.82240608 |
|  | rs62107261 | 0.0447316 | 2.13E-11 | -0.0259 | 0.004 | 805431 | 5.21E-05 | 41.92552089 |
|  | rs62181771 | 0.559642 | 4.47E-10 | 0.00989 | 0.002 | 805431 | 3.04E-05 | 24.45296428 |
|  | rs62250713 | 0.633201 | 1.63E-30 | -0.0189 | 0.002 | 805431 | 1.11E-04 | 89.30227825 |
|  | rs6265 | 0.196819 | 1.86E-22 | -0.0199 | 0.002 | 805431 | 1.23E-04 | 99.00225416 |
|  | rs6464024 | 0.437376 | 3.38E-14 | -0.0121 | 0.002 | 805431 | 4.54E-05 | 36.60240911 |
|  | rs6497840 | 0.713718 | 1.13E-12 | 0.0128 | 0.002 | 748334 | 5.47E-05 | 40.95989053 |
|  | rs6598539 | 0.512922 | 5.76E-12 | 0.011 | 0.002 | 780333 | 3.88E-05 | 30.24992247 |
|  | rs66680800 | 0.430418 | 1.13E-14 | -0.0125 | 0.002 | 805431 | 4.85E-05 | 39.062403 |
|  | rs6728726 | 0.83002 | 4.27E-19 | 0.0187 | 0.002 | 805431 | 1.09E-04 | 87.42228292 |
|  | rs71367545 | 0.161034 | 1.70E-10 | 0.0124 | 0.002 | 805431 | 4.77E-05 | 38.43990455 |
|  | rs71627577 | 0.119284 | 5.15E-12 | -0.017 | 0.002 | 805431 | 8.97E-05 | 72.24982059 |
|  | rs7224742 | 0.633201 | 1.02E-11 | -0.011 | 0.002 | 805431 | 3.76E-05 | 30.24992488 |
|  | rs72789627 | 0.134195 | 1.54E-13 | -0.0171 | 0.002 | 805431 | 9.08E-05 | 73.10231848 |
|  | rs7333559 | 0.791252 | 3.24E-13 | -0.014 | 0.002 | 805431 | 6.08E-05 | 48.99987833 |
|  | rs745570 | 0.49503 | 8.70E-11 | -0.0104 | 0.002 | 780334 | 3.47E-05 | 27.0399307 |
|  | rs763053 | 0.267396 | 6.71E-16 | -0.0149 | 0.002 | 799280 | 6.94E-05 | 55.50236112 |
|  | rs76608582 | 0.0457256 | 2.76E-13 | -0.0267 | 0.004 | 799016 | 5.58E-05 | 44.55551347 |
|  | rs7804551 | 0.163022 | 1.09E-12 | -0.0152 | 0.002 | 805431 | 7.17E-05 | 57.75985657 |
|  | rs78175438 | 0.11829 | 2.02E-11 | 0.0162 | 0.002 | 805431 | 8.15E-05 | 65.60983708 |
|  | rs7829715 | 0.560636 | 1.30E-13 | -0.0117 | 0.002 | 805431 | 4.25E-05 | 34.22241502 |
|  | rs7969559 | 0.681909 | 3.28E-10 | -0.011 | 0.002 | 805431 | 3.76E-05 | 30.24992488 |
|  | rs8067305 | 0.574553 | 4.95E-12 | 0.011 | 0.002 | 805431 | 3.76E-05 | 30.24992488 |
|  | rs846781 | 0.737575 | 4.80E-11 | -0.0115 | 0.002 | 805431 | 4.10E-05 | 33.0624179 |
|  | rs9323328 | 0.526839 | 7.25E-12 | -0.0108 | 0.002 | 805431 | 3.62E-05 | 29.15992759 |
|  | rs9375371 | 0.293241 | 1.41E-13 | 0.013 | 0.002 | 805431 | 5.25E-05 | 42.24989509 |
|  | rs951740 | 0.636183 | 3.78E-26 | 0.0172 | 0.002 | 805431 | 9.18E-05 | 73.95981635 |
|  | rs963354 | 0.704771 | 2.35E-10 | 0.0107 | 0.002 | 805431 | 3.55E-05 | 28.62242893 |
|  | rs9679319 | 0.56163 | 4.52E-23 | 0.0175 | 0.002 | 640245 | 1.20E-04 | 76.56226083 |
|  | rs9763225 | 0.26839 | 5.38E-12 | 0.0139 | 0.002 | 624207 | 7.74E-05 | 48.30234524 |
|  | rs993700 | 0.767396 | 1.00E-13 | -0.0141 | 0.002 | 805431 | 6.17E-05 | 49.70237658 |
|  | rs9987376 | 0.569583 | 1.02E-17 | -0.0137 | 0.002 | 805431 | 5.83E-05 | 46.92238348 |
|  |  |  |  |  |  |  |  |  |
| alcohol consumption | rs10236149 | 0.137177 | 4.40E-10 | -0.0163 | 0.003 | 666978 | 4.43E-05 | 29.52102259 |
|  | rs10276148 | 0.530815 | 1.19E-11 | 0.0117 | 0.002 | 666978 | 5.13E-05 | 34.22239738 |
|  | rs10743083 | 0.815109 | 1.64E-10 | -0.015 | 0.002 | 611077 | 9.20E-05 | 56.2498159 |
|  | rs11692435 | 0.083499 | 7.20E-11 | 0.0191 | 0.003 | 653991 | 6.20E-05 | 40.53432048 |
|  | rs11860773 | 0.184891 | 1.04E-12 | -0.0157 | 0.002 | 665054 | 9.26E-05 | 61.62231468 |
|  | rs11940694 | 0.611332 | 9.51E-56 | 0.0279 | 0.002 | 666978 | 2.92E-04 | 194.6019165 |
|  | rs11943397 | 0.62326 | 1.23E-11 | 0.0122 | 0.002 | 666978 | 5.58E-05 | 37.20988842 |
|  | rs1229984 | 0.971173 | 1.00E-200 | 0.193 | 0.005 | 660728 | 2.25E-03 | 1489.95549 |
|  | rs1260326 | 0.589463 | 3.16E-46 | 0.0254 | 0.002 | 666978 | 2.42E-04 | 161.2895164 |
|  | rs13024996 | 0.359841 | 5.18E-14 | -0.0136 | 0.002 | 666978 | 6.93E-05 | 46.23986134 |
|  | rs13107325 | 0.0795229 | 2.86E-28 | -0.0393 | 0.004 | 666978 | 1.45E-04 | 96.53033554 |
|  | rs13236841 | 0.235586 | 1.92E-12 | -0.014 | 0.002 | 666978 | 7.35E-05 | 48.99985307 |
|  | rs13332432 | 0.293241 | 1.64E-11 | 0.0129 | 0.002 | 665054 | 6.26E-05 | 41.60237489 |
|  | rs147431626 | 0.249503 | 7.52E-32 | -0.0294 | 0.002 | 463556 | 4.66E-04 | 216.0890677 |
|  | rs16854020 | 0.11829 | 6.28E-13 | 0.0189 | 0.003 | 666978 | 5.95E-05 | 39.68988099 |
|  | rs2049045 | 0.192843 | 2.24E-11 | -0.0151 | 0.002 | 666978 | 8.55E-05 | 57.00232907 |
|  | rs2424645 | 0.348907 | 1.15E-10 | -0.0117 | 0.002 | 666978 | 5.13E-05 | 34.22239738 |
|  | rs2533126 | 0.471173 | 1.98E-14 | 0.0133 | 0.002 | 666978 | 6.63E-05 | 44.22236739 |
|  | rs28601761 | 0.409543 | 1.57E-10 | 0.0112 | 0.002 | 666978 | 4.70E-05 | 31.35990596 |
|  | rs28616142 | 0.418489 | 2.99E-10 | 0.0112 | 0.002 | 666977 | 4.70E-05 | 31.35990596 |
|  | rs28680958 | 0.22167 | 1.08E-10 | -0.0137 | 0.002 | 665054 | 7.05E-05 | 46.92235889 |
|  | rs28732378 | 0.718688 | 1.55E-23 | -0.0197 | 0.002 | 666978 | 1.45E-04 | 97.02220907 |
|  | rs28929474 | 0.0168986 | 3.28E-14 | -0.0489 | 0.006 | 666978 | 9.96E-05 | 66.42230083 |
|  | rs4337071 | 0.362823 | 5.59E-17 | -0.0151 | 0.002 | 666978 | 8.55E-05 | 57.00232907 |
|  | rs4916723 | 0.413519 | 2.74E-10 | -0.0111 | 0.002 | 666978 | 4.62E-05 | 30.80240764 |
|  | rs530916 | 0.56163 | 4.22E-10 | 0.011 | 0.002 | 650123 | 4.65E-05 | 30.24990694 |
|  | rs55932213 | 0.701789 | 1.61E-10 | 0.0127 | 0.002 | 666978 | 6.05E-05 | 40.32237909 |
|  | rs61934664 | 0.463221 | 8.96E-12 | -0.0118 | 0.002 | 666978 | 5.22E-05 | 34.80989562 |
|  | rs62305763 | 0.0924453 | 1.15E-28 | 0.0303 | 0.003 | 666978 | 1.53E-04 | 102.0096941 |
|  | rs6739804 | 0.642147 | 2.85E-13 | -0.0136 | 0.002 | 666978 | 6.93E-05 | 46.23986134 |
|  | rs75199129 | 0.0387674 | 8.23E-12 | -0.0283 | 0.004 | 666978 | 7.50E-05 | 50.0554749 |
|  | rs8020892 | 0.737575 | 2.06E-12 | -0.0134 | 0.002 | 666978 | 6.73E-05 | 44.88986539 |
|  | rs838145 | 0.581511 | 5.62E-21 | -0.0164 | 0.002 | 665346 | 1.01E-04 | 67.23979788 |
