# Supplementary material for: Impact of smoking and alcohol consumption on telomere length: A univariable and multivariable Mendelian randomization study
Source: Medicine (Baltimore). 2025 Sep 26;104(39):e44685. doi: 10.1097/MD.0000000000044685 (PMC13593225; doi:10.1097/MD.0000000000044685)
Supplement: Supplementary file 2 [file medi-104-e44685-s002.docx]

**Supplemental Figure 1.** Leave-one-out analysis of the causal effect of smoking initiation on telomere length. MR = Mendelian randomization.

**Supplemental Figure 2.** Funnel plots of the causal effect of smoking initiation on telomere length. MR = Mendelian randomization; SE = standard error.

**Supplemental Figure 3.** Leave-one-out analysis of the causal effect of alcohol consumption on telomere length. MR = Mendelian randomization.

**Supplemental Figure 4.** Funnel plots of the causal effect of alcohol consumption on telomere length. MR = Mendelian randomization; SE = standard error.
